# Effect of microbial siderophores on mammalian non-malignant and malignant cell lines

**DOI:** 10.1186/s12906-017-1657-8

**Published:** 2017-03-09

**Authors:** Karuna Gokarn, Vishwas Sarangdhar, Ramprasad B. Pal

**Affiliations:** 1 Department of Microbiology, Sir Hurkisondas Nurrotumdas Medical Research Society, Mumbai, 400 002 India; 2 Caius Research Laboratory, St. Xavier’s College, Mumbai, 400 001 India

**Keywords:** Siderophores, Exochelin-MS, Mycobactin S, Deferoxamine B, Mammalian cell lines, MTT assay

## Abstract

**Background:**

Iron is a vital nutrient for all cells, and malignant cells have a higher requirement for the metal due to their rapid multiplication. Bacterial siderophores can be used to reduce free ferric ion concentration from the cellular environment.

**Methods:**

In the present study, we have evaluated effect of three siderophores – exochelin-MS, mycobactin S and deferoxamine B on the proliferation of mammalian cell lines using MTT assay.

**Results:**

These siderophores caused a significant decrease in the viability of malignant cells, without significantly affecting non-malignant cells.

**Conclusions:**

Based on these results, we suggest that iron-chelation therapy could be explored as an adjunctive therapeutic option against cancer along with other therapies.

## Background

Iron is a vital nutrient for all cells. In eukaryotes, the highly iron-dependent activity of ribonucleotide reductase is essential for cell division [1]. Under iron-deficient conditions, cells cannot transit from G1-phase to S-phase in the cell cycle. Neoplastic cells have a higher requirement for iron than normal cells as they proliferate at a faster rate. Therefore, iron depletion by iron-chelating agents may deprive the rapidly dividing malignant cells of the DNA precursors required for replication, resulting in the inhibition of their proliferation [2, 3].

Bacterial iron chelators termed siderophores can be used for reducing free iron concentration in the cellular environment. Growing microbial cells in iron-deficient laboratory growth media allows isolation and characterization of the siderophores.

Exochelin-MS (Exo-MS) is a water-soluble siderophore and mycobactin S (MBS) is a lipid-soluble siderophore produced by *Mycobacterium smegmatis*. Water-soluble deferoxamine mesylate B (DFO-B), originally from *Streptomyces pilosus*, is now commercially available.

Effect of Exo-MS, MBS and DFO-B were evaluated individually on malignant and non-malignant cell lines of murine and human origin. The concept tested here was whether iron deprivation by microbial siderophores affects multiplication of mammalian cells. This was assessed in vitro by MTT assay.

## Methods

For the production of siderophores from *M. smegmatis* mc^2^155, iron-deficient minimal medium was used [4, 5]. The culture of *M. smegmatis* mc^2^155 was inoculated and incubated at 37 °C for 10 days on the shaker at 200 rpm for Exo-MS production; whereas, for the production of MBS, the culture flasks were incubated at 37 °C for 10 days under static conditions.

### Extraction, detection and purification of Exo-MS

- Extraction of Exo-MS:

The10-day old culture broth of *M. smegmatis* was centrifuged at 10,000 rpm for 10 min to pellet the cells. The supernatant, in batches of 100 mL was freeze-dried, subsequently redissolved in 10 mL of distilled water and the pH was adjusted to 3.5. Then, it was saturated with ammonium sulfate (76 g/100 mL), and kept at 4 °C overnight. Next day, the solution was centrifuged and the supernatant was transferred to a 50 mL tube and treated with 2.0 mL of benzyl alcohol. The aqueous and the solvent phases were mixed vigorously, centrifuged and the upper phase of benzyl alcohol fraction was collected in a clean test tube. This extraction step was repeated three times and all the benzyl alcohol fractions were pooled [6]. The pooled extract was mixed with 8.0 mL of diethyl ether for partitioning the water soluble Exo-MS. Exo-MS was extracted with 1.0 mL of distilled water. The solvent-water mixture was centrifuged, and the lower aqueous phase was transferred to a clean tube. This step too was repeated thrice with 1.0 mL distilled water each time. All the aqueous fractions were pooled and washed with diethylether to remove traces of benzyl alcohol. This aqueous Exo-MS preparation was concentrated by freeze-drying which also removed any residual diethyl ether.

(b) Purification and Detection of Exo-MS:

The Exo-MS extract was purified on a column of neutral grade alumina. When the column was eluted with petroleum spirit (bp = 60 to 80 °C), the Exo-MS was firmly bound to the alumina, whereas the faster-migrating compounds were washed away with no retardation. Hydrophobic impurities were removed from the alumina-bound Exo-MS by sequential washes with cyclohexane, toluene, diethyl ether, ethyl acetate, and chloroform; all the washes were discarded. The adsorbed Exo-MS was then eluted from the column with the methanol-formic acid (4:1, v/v) mixture [7]. The eluate was concentrated by freeze-drying and neutralized to pH 6.5. The concentration of this Exo-MS preparation was determined by UV spectrophotometer.

The Exo-MS preparation was also subjected to high-resolution liquid chromatography/mass spectrometry (HR-LC/MS) using Agilent Technologies Q-TOF Mass Spectrometer system at Sophisticated Analytical Instrument Facility, Indian Institute of Technology Bombay, India. The solvent system was 95 mL of 0.1% of Trifluoroacetic acid (TFA) in water with 5 mL of 0.1% TFA in 90% Acetonitrile (ACN) for 22 min. Then, 5 mL of 0.1% of Trifluoroacetic acid (TFA) in water with 95 mL of 0.1% TFA in 90% Acetonitrile (ACN) for 9 min. This was followed by the first solvent mixture for 5 min. The flow-rate was 0.4 mL/min for 35 min with maximum pressure of 1200 bar.

Two dimensional nuclear magnetic resonance - total correlation spectroscopy (2D-NMR-TOCSY) was carried out at National Facility for High-Field NMR, Tata Institute of Fundamental Research, Mumbai, India. The solvent system was 10% D_2_O and 90% water, which was run for 2 h on 500 MHz instrument.

### Extraction, detection and purification of MBS

- Extraction of MBS:

The 10-day old culture broth of *M. smegmatis* mc^2^155 was centrifuged and the cell pellet was transferred to a flask containing 100 mL of ethanol and kept overnight in the refrigerator. Next day, this extract was mixed with equal volume of chloroform. Sufficient water was added to this mixture to form two layers. The upper aqueous layer was discarded, and the lower chloroform layer containing MBS was washed with water, dehydrated over anhydrous Na_2_SO_4_ and evaporated to dryness. The dried residue was extracted with methanol at room temperature. The methanol-soluble MBS was thus separated from the other insoluble impurities [8].

(b) Purification and Detection of MBS:

Methanol-soluble MBS was evaporated to dryness and then dissolved in a minimum amount of tertiary butanol : cyclohexane (1:1, v/v) ratio. After dissolving the residue, cyclohexane was added further to make up the volume to 100 mL. This solution was then adsorbed onto neutral grade alumina in a beaker and washed with 50 mL of cyclohexane. The wash was discarded. The adsorbed material was then sequentially washed with 50 mL each of petroleum ether, toluene and diethyl ether, which were also discarded. Finally, MBS was eluted using chloroform: acetone mixture (1:1, v/v) [9]. This eluate was evaporated to dryness again, dissolved in absolute ethanol and subjected to Sephadex LH 20 column chromatography. MBS was eluted using absolute ethanol. UV spectrophotometer was used to determine the concentration of this MBS preparation.

Reverse-phase HPLC was used to confirm the purity of MBS after adding 10 μL of 10% ethanolic ferric chloride to 100 μL of the MBS preparation to get ferri-MBS, which was carried out on Waters Xterra MS MS system at Bombay College of Pharmacy, Mumbai, India. The gradient solvent system used for HPLC was methanol: water (80:20, v/v) which was passed through a C_18_ column for 30 min and then with 100% methanol for the next 15 min. The column used was, C_18_, 5 μm, 4.6 × 100 mm at the flow-rate of 1.5 mL per min. The eluted peaks were determined by monitoring the absorbance of the sample at *A*_220nm_ and *A*_450nm_.

### Purity analysis of DFO-B

DFO-B manufactured by Novartis, Switzerland, and obtained as 500 mg dry powder was dissolved in 2 mL sterile water at a concentration of 250 mg /mL. For HPLC, ferrioxamine-B was prepared by mixing 10 μL of 10% aqueous ferric chloride to 100 μL of 1.0 mg/mL DFO-B. Gradient solvent system used was 0.1% Trifluoroacetic acid (TFA) in water for 10 min, then equal volumes of 0.1% aqueous TFA and acetonitrile (ACN) (1:1, v/v) for 30 min and 0.1% TFA in water for 10 min. The column used was C18, 5 μm, 4.6 × 100 mm, at a flow-rate of 1.5 mL per min. The peaks were monitored by measuring the absorbance at 420 nm. This analysis was carried out on Waters Xterra MS MS system at Bombay College of Pharmacy, Mumbai, India.

### Determination of the effect of Exo-MS, DFO-B, and MBS on mammalian cell lines

The effect of Exo-MS, DFO-B, and MBS was evaluated using malignant and non-malignant mammalian cell lines. A working stock solution of DFO-B at 100 mg/mL was prepared from 250 mg/mL stock solution before use. Exo-MS was dissolved in water and MBS in methanol for use.

Cell Lines: The cell lines used were as follows:

- Adherent malignant cell lines - RAW 264.7 (murine macrophage), MCF-7 (human breast), HEPG2 (human liver);
- Non-adherent malignant cell line – K 562 (human leukemia);
- Adherent non-malignant cell lines - NIH/3 T3 (mouse embryonic fibroblast) and HEK 293 (human embryonic kidney).

The cell lines were procured from NCCS, Pune, India. They were maintained in T-25 flasks in Dulbecco’s Modified Eagle’s Medium (DMEM) with 10% Fetal Bovine serum (FBS) at 37 °C in a CO_2_ incubator with 5% CO_2_ in humidified air.

Cell culture growth medium: DMEM, FBS and Trypsin-EDTA were obtained from Cell Clone (Genetix Biotech Asia, India).

Cell viability assay using MTT reagent: MTT {3-(4, 5-dimethylthiazolyl-2)-2, 5-diphenyltetrazolium bromide} assay was used to determine the viability of cells. This assay is a useful quantification method which measures metabolic activity of cells [10, 11]. Metabolically active cells reduce yellow-colored tetrazolium and form purple crystals of formazan. These crystals upon solubilization with DMSO (dimethyl sulfoxide) were quantified by measuring the absorbance at 570 nm and 655 nm using a microplate reader.

All the cells used in the study were seeded in triplicates at a concentration of 6 × 10^4^ cells/mL of complete DMEM in 12-well plates (total volume was 1.0 mL) and incubated for 24 h. Next day, different concentrations of each of the siderophores – Exo-MS, DFO-B, and MBS were added and incubated for another 24 h. Since MBS was prepared in methanol, methanol control was also maintained, where the cells were incubated with only methanol. The assay contained:

- Blank wells with only medium;
- Control wells with untreated cells;
- Control wells with cells treated with sodium azide;
- Test wells with cells treated with the siderophores.

Next day, from adherent cells, spent medium was discarded, and 1.0 mL of MTT reagent in DMEM was added to the wells and incubated at 37 °C for 4 h. For the non-adherent cell line, the entire suspension was centrifuged in a 1.5 mL microfuge tube to pellet the cells. The supernatant was carefully poured out, and the cell pellet was then resuspended in 1.0 mL of MTT reagent, reloaded in the same wells and incubated for 4 h at 37 °C. At the end of the incubation period, 1.0 mL of 100% DMSO was added to solubilize the visible formazan crystals. The plates were placed on a rocker for 20 min, and the absorbance of each well was measured at 570 nm and 655 nm using Epoch 2 (Biotek Instruments, USA) instrument.

MTT assays for every cell line were performed as three independent experiments, each in triplicate. Statistical analytical software - GraphPad Prism 7.01 and SigmaStat 4.0 – were used to compare the effect of Exo-MS, DFO-B and MBS on each cell line. The readings of control wells containing untreated cells were considered as 100% survival. The readings of control wells containing cells treated with 0.1% sodium azide were considered as no survival.

## Results

### Purity analysis of siderophores

**Fig. 1:**
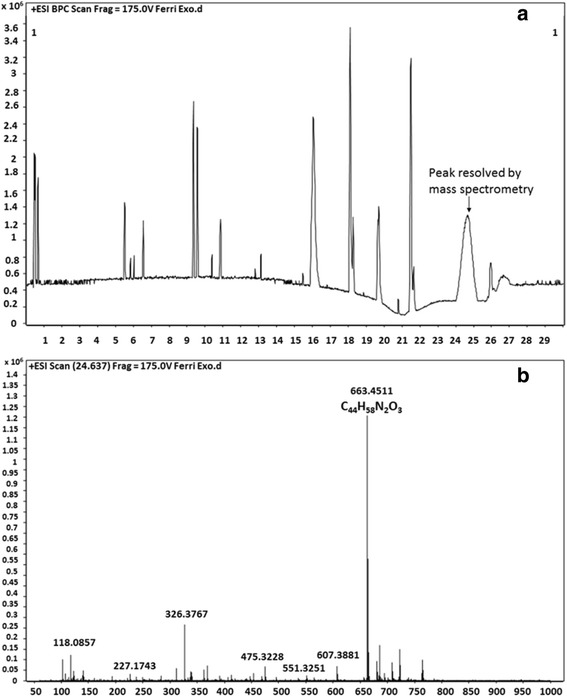
HR-LC/MS of ferri-exochelin-MS: Exo-MS preparation was analyzed by injecting 5 μL volume in the column. Agilent system Q-TOF Mass Spectrometer was used. The solvent system was 95 mL of 0.1% of Trifluoroacetic acid (TFA) in water with 5 mL of 0.1% TFA in 90% Acetonitrile (ACN) for 22 min. Then, 5 mL of 0.1% of Trifluoroacetic acid (TFA) in water with 95 mL of 0.1% TFA in 90% Acetonitrile (ACN) for 9 min. This was followed by the first solvent mixture for 5 min. The flow-rate was 0.4 mL/min for 35 min with maximum pressure of 1200 bar. **a** High-resolution liquid chromatogram: The peak seen between 24.036 and 25.271 showed the maximum area which was used for further resolution and detection by MS. **b** Mass spectrometry of the peak at 24 min from (**a**). Peaks at 663.45 and 326.37 are due to MH^+^ and MH2^2+^, respectively

i) The HR-LC/MS of ferri-Exo-MS showed many peaks at 420 nm from 0 to 30 min (Fig. 1a and Table 1). The major peak was between 24 and 25 min. The area under this curve was considered to be 100% and the relative concentrations of the other peaks were measured.

**Table 1:** HR-LC/MS integration peak list of Exo-MS

Peak	Start	RT	End	Height	Area	Area %	AreaSumPercent	BasePeakMz
1	0.423	0.474	0.574	1605457	7925924	20.49	5.51	150.1117
2	0.641	0.691	0.741	1260173	3247945	8.4	2.26	139.0495
3	5.433	5.517	5.617	929895	3587468	9.27	2.5	453.3415
4	9.274	9.357	9.441	2115277	5968077	15.43	4.15	568.2943
5	9.508	9.574	9.675	1806352	5584557	14.44	3.89	383.1507
6	15.853	16.054	16.438	2043093	25462871	65.83	17.72	326.377
7	18.024	18.108	18.225	3059381	14141487	36.56	9.84	311.1631
8	19.56	19.711	19.945	1145362	9522103	24.62	6.63	338.34
9	21.397	21.497	21.614	2927045	14662592	37.91	10.2	413.2648
10	24.036	24.654	25.271	1030920	38679784	100	26.91	663.451
  The MS-MS results showed a significant peak corresponding to a molecular weight of 663 indicating presence of Exo-MS (Fig. 1b). The second major peak had a molecular weight of 326 indicating the presence of doubly charged Exo-MS.
  For 2D NMR-TOCSY of Exo-MS, the concentration used was 800 μg/mL. The analysis was done on a 500 MHz instrument with 10% D2O in water for 2 h. The chromatogram obtained showed the spin systems of threonine, β-alanine and γ1 β-ornithine (Fig. 2).

**Fig. 2:**
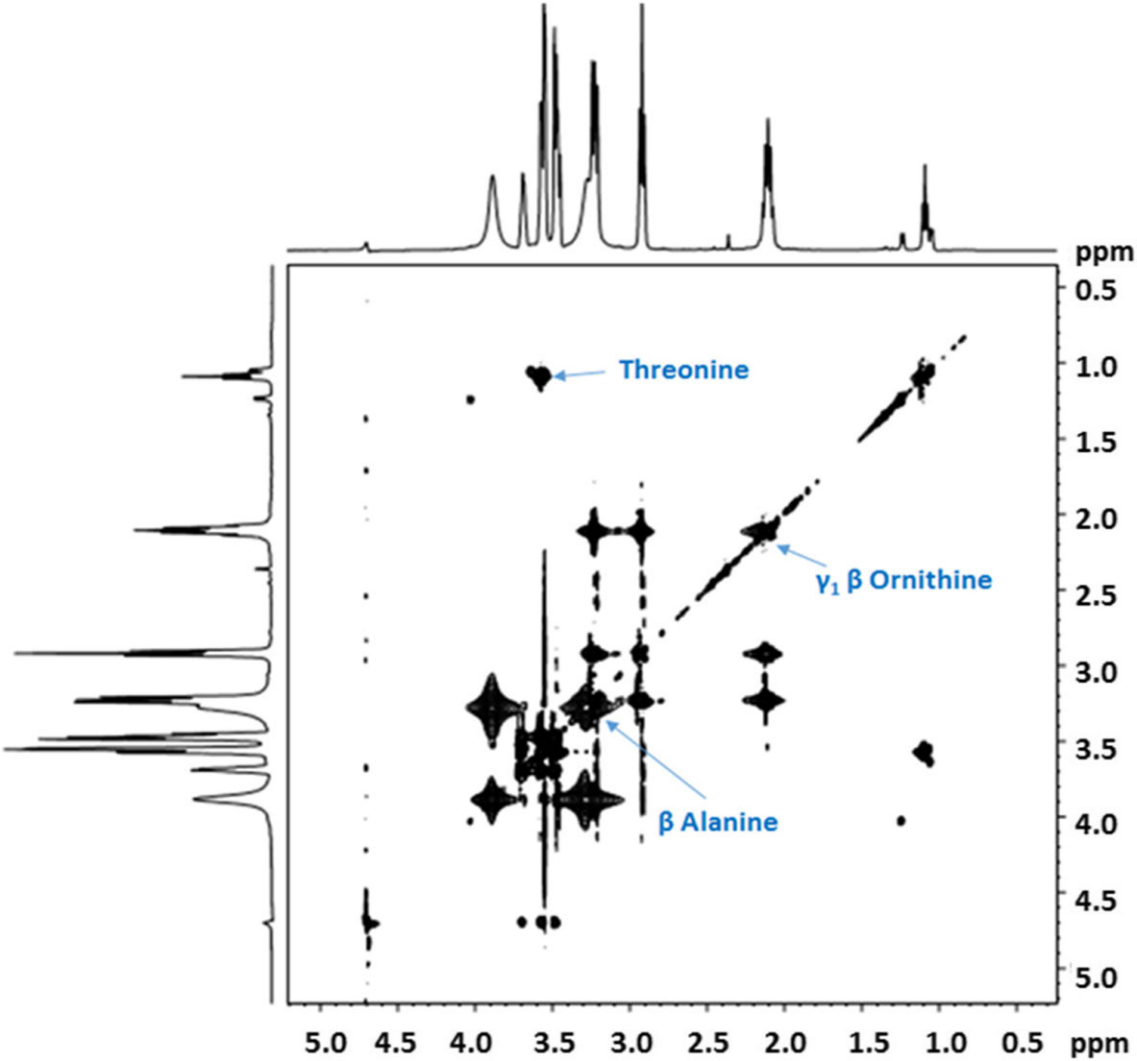
The two-dimensional nuclear magnetic resonance spectroscopy 2D NMR-TOCSY of ferri-Exo-MS. Exo-MS at a concentration of 800 μg/mL was analyzed on a 500 MHz instrument with 10% D2O in water for 2 h. The spin systems of threonine, β-alanine and γ1 β-ornithine are seen
ii) HPLC of ferri-MBS: HPLC analysis of ferri-MBS showed peaks in order of increasing acyl-chain length, which were visualized as seven peaks at 450 nm (Fig. 3a), when 10 μL of 4 mg/mL concentration of MBS was used. Three peaks were seen between 5 and 10 min, one major peak between 10 and 15 min along with a minor peak. The sixth and seventh peaks were seen between 20 and 25 min and between 35 and 40 min, respectively. The absorbance of ferri-MBS at 220 nm (Fig. 3b) was measured to know the extent of purity of the MBS preparation. The peaks at 220 nm were at the same positions as that seen at 450 nm.

**Fig. 3:**
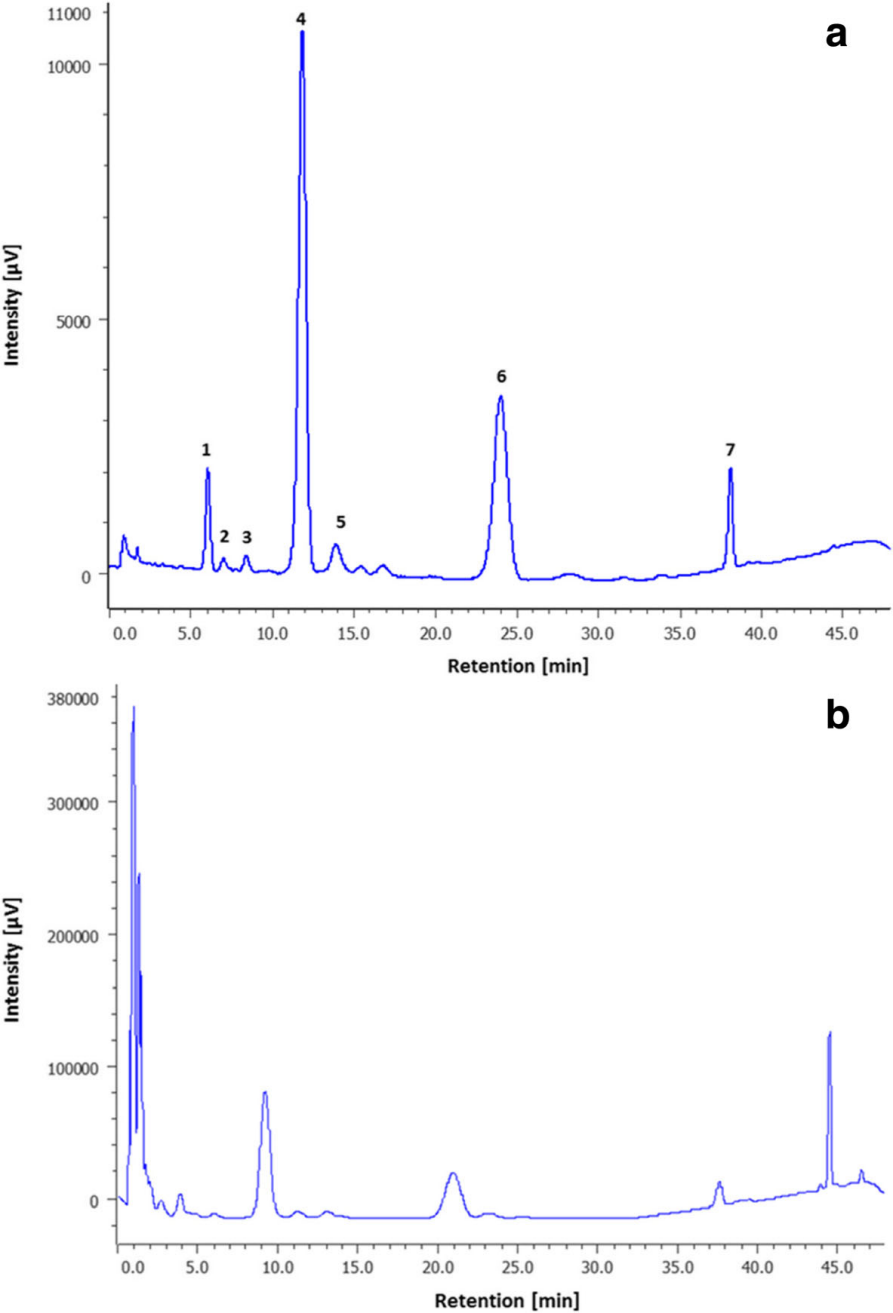
HPLC of pure ferri-mycobactin S. MBS at 4 mg/mL concentration was analyzed by injecting 10 μL volume in Waters Xterra MS MS HPLC system. The column used was C_18_, 5 μm, 4.6 × 100 mm at the flow-rate of 1.5 mL per min. Solvent system was methanol: water (80:20, v/v) for 30 min followed by 100% methanol for 15 min. (**a**) at 450 nm; (**b**) 220 nm. Seven peaks of different intensities were observed depending upon the acyl chain length that varies from 9 to 19 carbons; the shortest chain being eluted out first and the longest last
iii) The HPLC chromatogram of ferrioxamine B showed a single sharp peak between 15 and 20 min (Fig. 4). Gradient Trifluoroacetic acid-Acetonitrile (TFA-ACN) solvent system was used for analysis of 10 μL of 1.0 μg/mL concentration of ferrioxamine B.

**Fig. 4:**
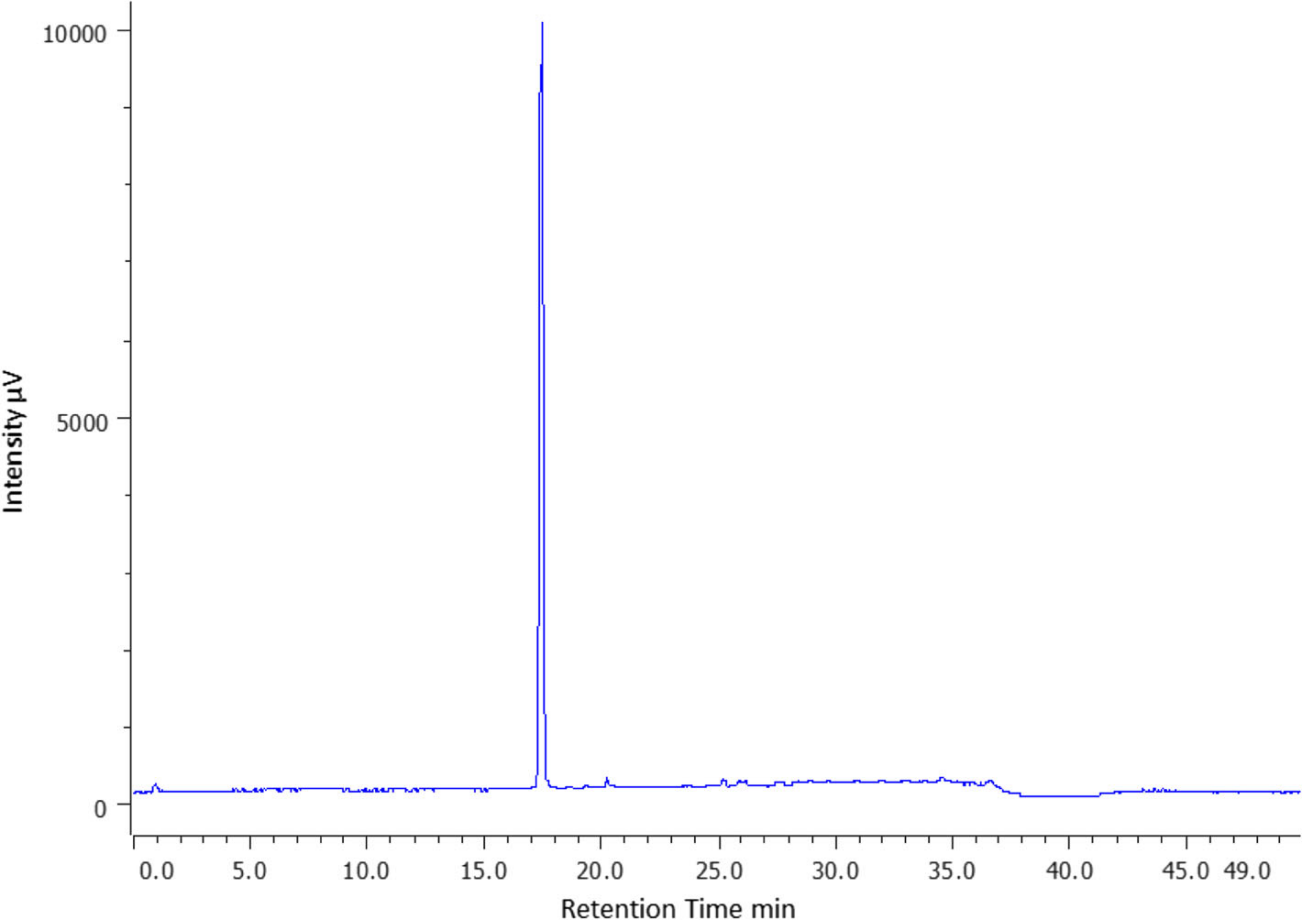
HPLC of ferrioxamine B. Ferrioxamine B at 1.0 μg/mL concentration was analysed by injecting 10 μL in Waters Xterra MS MS HPLC system. The column used was C18, 5 µm, 4.6 x 100 mm, at a flow-rate of 1.5 mL per min. Solvent system was 0.1% trifluoroacetic acid (TFA) in water (v/v) for first 10 min, followed by equal volumes of 0.1% aqueous TFA and acetonitrile (1:1 v/v) for the next 30 min. And then 0.1% TFA in water (v/v) for the next 10 min, peaks monitored at 420nm. The chromatogram showed a single peak between 15-20 min

To determine the concentration of purified siderophores, *A*_420nm_ of Exo-MS and *A*_450nm_ of MBS were measured using UV-VIS spectrophotometer. As per Ratledge and Ewing [12], the extinction coefficients are:

- Exo-MS: *E*_1*cm*_^1%^ = 15.8 in water;
- MBS: *E*_1*cm*_^1%^ = 43 in water;

Using the above correlations, the concentrations of siderophores purified for use in these assays were Exo-MS = 19 mg/mL and MBS = 4 mg/mL.

### Effect of Exo-MS, MBS, and DFO-B on mammalian cell lines

The MTT reagent exhibited negligible background absorbance values in blank wells without the cells. The absorbance values of untreated cells were considered as 100%. The absorbance reading from each well, (*A*_570nm_ - *A*_655nm_) was calculated, and then the following formula was used to determine the percent survival;

$$ \mathrm{Percent}\ \mathrm{Survival}=\kern0.5em \left[\frac{A_{\mathrm{treated}} - {A}_{\mathrm{blank}}}{A_{\mathrm{control}}-{A}_{\mathrm{blank}}}\right]\times 100 $$

The intensity of purplish pink colour produced is directly proportional to the number of viable cells. The readings of control wells containing untreated cells which were considered as 100% survival showed a dark purple colour. The readings of control wells containing cells treated with 0.1% sodium azide showed either very pale purple colour or were colourless (data not shown).

**Fig. 5:**
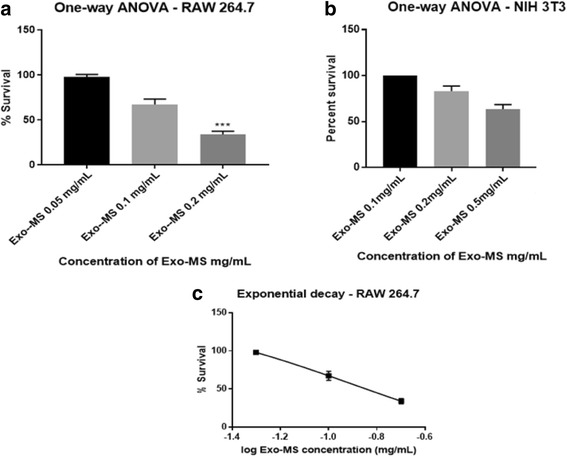
Effect of Exo-MS on murine cell lines RAW 264.7 and NIH/3 T3. The results of MTT assay were plotted using statistical analytical software - GraphPad Prism 7.01 and SigmaStat 4.0. The readings of control wells containing untreated cells were considered as 100% survival. **a** A statistically significant decrease in the percent survival of macrophage cancer cell line RAW 264.7 was seen at Exo-MS concentrations at 0.2 mg/mL (*P* <0.001). **b** No statistically significant reduction in the percent survival of normal mouse fibroblast cell line, NIH/3 T3 was seen at Exo-MS concentrations up to 0.5 mg/mL. **c** IC_50_ of Exo-MS = 0.128 mg/mL for RAW 264.7

Murine cell lines - RAW 264.7 and NIH/3 T3: A statistically significant decrease in the percent survival of RAW 264.7 was seen at Exo-MS concentrations of 0.2 mg/mL, where percentage of viable cells was 34% (Fig. 5a). No significant effect was seen on normal mouse fibroblast cell line NIH/3 T3 at Exo-MS concentrations up to 0.5 mg/mL. Percent cell survival was 100, 83 and 67% at 0.1 mg/mL, 0.2 mg/mL and 0.5 mg/mL, respectively (Fig. 5b). Figure 5c shows the IC_50_ of Exo-MS for RAW 264.7 which was 0.128 mg/mL.

**Fig. 6:**
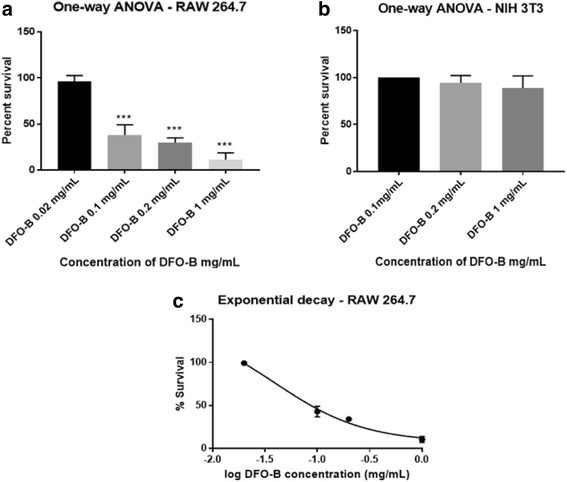
Effect of DFO-B on murine cell lines RAW 264.7 and NIH/3 T3. The results of MTT assay were plotted using statistical analytical software - GraphPad Prism 7.01 and SigmaStat 4.0. The readings of control wells containing untreated cells were considered as 100% survival. **a** A statistically significant decrease in the percent survival of macrophage cancer cell line RAW 264.7 was seen at DFO-B concentrations up to 1.0 mg/mL (*P* <0.001). **b** No statistically significant decrease in the percent survival of normal mouse fibroblast cell line, NIH/3 T3 was seen at DFO-B concentrations up to 1.0 mg/mL. **c** IC_50_ of DFO-B for RAW 264.7 was = 0.366 mg/mL

A statistically significant decrease in the percent survival of RAW 264.7 was seen at DFO-B at concentrations up to 1.0 mg /mL, where percent cell survival was 38, 30 and 11% at 0.1 mg/mL, 0.2 mg/mL and 1 mg/mL, respectively (Fig. 6a). No significant effect was seen on normal mouse fibroblast cell line NIH/3 T3 at DFO-B concentrations up to 1.0 mg/mL. Percent cell survival was 100, 94 and 87% at 0.1 mg/mL, 0.2 mg/mL and 1 mg/mL, respectively (Fig. 6b). Figure 6c shows that the IC_50_ of DFO-B for RAW 264.7 which was 0.366 mg/mL.

#### Human Cell Lines – K 562, MCF-7, HEK 293

**Fig. 7:**
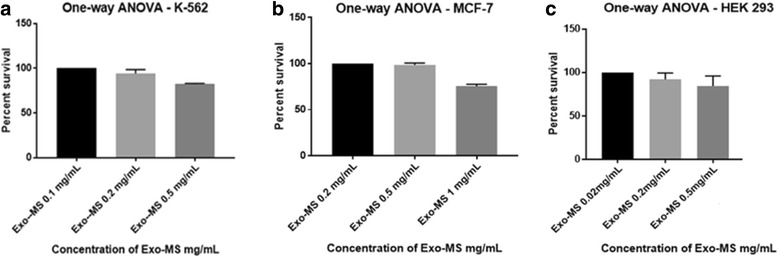
Effect of Exo-MS on human cell lines K 562, MCF-7 and HEK 293. The results of MTT assay were plotted using statistical analytical software - GraphPad Prism 7.01 and SigmaStat 4.0. The readings of control wells containing untreated cells were considered as 100% survival. No statistically significant decrease in the percent survival was observed for (**a**) human leukemia cell K 562 at Exo-MS concentrations up to 0.5 mg/mL (**b**) breast cancer cell line MCF-7 at Exo-MS concentrations up to 1.0 mg/mL and (**c**) normal embryonic kidney cell line HEK 293 at Exo-MS concentrations up to 0.5 mg/mL

No statistically significant decrease in the percent survival of K 562 and MCF-7 at Exo-MS concentrations at 0.5 mg/mL and 1.0 mg/mL, respectively, was observed. For K 562, percent survival was 94 and 82%, respectively (Fig. 7a) and for MCF-7, it was 100 and 88.5%, respectively (Fig. 7b). Similarly, no significant decrease in the percent survival of HEK 293 at Exo-MS concentrations up to 1.0 mg/mL was observed. Percent cell survival at Exo-MS concentrations 0.2 mg/mL and 0.5 mg/mL for HEK 293 was 92 and 90%, respectively (Fig. 7c).

**Fig. 8:**
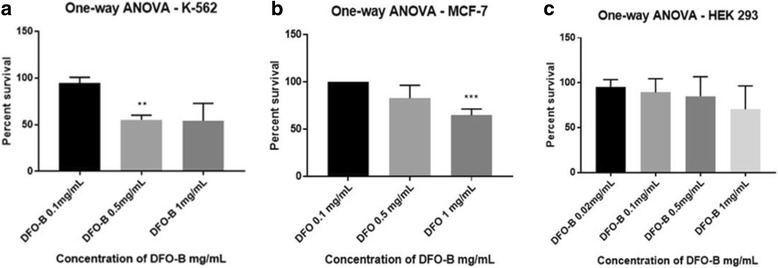
Effect of DFO-B on human cell lines K 562, MCF-7 and HEK 293. The results of MTT assay were plotted using statistical analytical software - GraphPad Prism 7.01 and SigmaStat 4.0. The readings of control wells containing untreated cells were considered as 100% survival. **a** For human leukemia cell K 562, a statistically significant reduction in cell survival at DFO-B concentration of 0.5 mg/mL. **b** A statistically significant decrease in cell survival was seen with breast cancer cell line MCF-7 at DFO-B concentration of 1.0 mg/mL was observed (*P* <0.01). **c** No statistically significant decrease in the percent survival of normal embryonic kidney cell line HEK 293 was observed at DFO-B concentration upto 1.0 mg/mL

The percent viability of K 562, MCF-7, HEK 293 in the presence of DFO-B. For K 562, a statistically significant reduction in cell survival at DFO-B concentration of 0.5 mg/mL was observed, (*P* <0.01). Percent cell survival at DFO-B concentrations – 0.5 mg/mL and 1 mg/mL for K 562 was 55 and 54%, respectively (Fig. 8a).

There was a statistically significant reduction in the percent cell survival of breast cancer cell line MCF-7 at DFO-B concentration of 1.0 mg/mL, (*P* <0.01). Percent cell survival at DFO-B concentration of 1 mg/mL for MCF-7 was 62% (Fig. 8b).

There was no statistically significant decrease in the percent survival of HEK 293. Percent cell survival at DFO-B concentrations – 0.5 mg/mL and 1 mg/mL for HEK 293 was 92 and 88%, respectively (Fig. 8c).

**Fig. 9:**
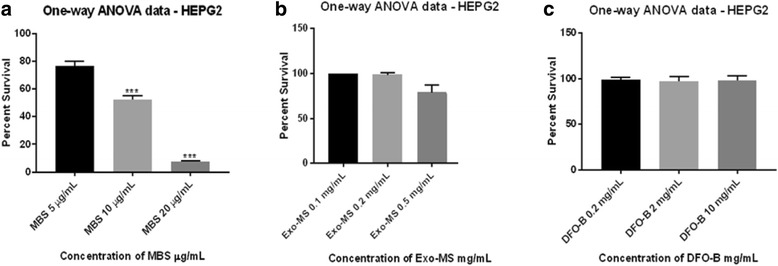
Effect of MBS, Exo-MS, and DFO-B on human liver cancer cell line HEPG2. The results of MTT assay were plotted using statistical analytical software - GraphPad Prism 7.01 and SigmaStat 4.0. The readings of control wells containing untreated cells were considered as 100% survival. **a** A statistically significant decrease in viability of the cells at MBS concentrations from 10 μg/mL onwards was observed (*P* <0.001). **b** and **c** No statistically significant difference was observed in percent survival of HEPG2 at the Exo-MS and DFO-B concentrations up to 0.5 mg/mL and 10 mg/mL, respectively

There was a statistically significant decrease in viability of HEPG2 cells at MBS concentrations from 10 μg/mL onwards (*P* <0.001). Percent cell survival at MBS concentrations of 5 μg/mL, 10 μg/mL and 20 μg/mL were 92.4, 48 and 23%, respectively (Fig. 9a). Methanol did not have any statistically significant effect on the cells since 95% survival of cells was observed (data not shown). Figure 9b and c show that there was no statistically significant decrease in percent survival of HEPG2 at the Exo-MS and DFO-B concentrations as high as 1 mg/mL and 10 mg/mL, respectively.

## Discussion

HPLC and HR-LC/MS analysis confirmed the presence of Exo-MS with some impurities. NMR technique not being sensitive, low concentrations of Exo-MS could not allow a clear correlation in TOCSY, though threonine, β-alanine and γ_1_β-ornithine could be deciphered.

HPLC analysis of ferri-MBS indicated about 98% purity. When the siderophore is complexed with iron, the red colored complex shows maximum absorbance at 450 nm, whereas the peptide bonds of MBS have an absorbance maximum at 220 nm. The peaks seen at A_220nm_ corresponded with the peaks observed at A_450 nm_ indicating pure MBS preparation. These were similar to the peaks obtained for MBS by Ratledge and Ewing [13].

Only one peak was obtained in the HPLC analysis of ferrioxamine-B indicating it was pure.

To be used for iron-depletion from microbial environments, siderophores need to be in a desferri form and not saturated with iron. Therefore, desferri form of siderophores were obtained using the isolation protocols for Exo-MS and MBS, but without the addition of iron during the entire isolation process. Iron was added to small aliquots only for determining purity and concentration of the siderophores.

### Effect of Exo-MS, MBS, and DFO-B on mammalian cell lines

Non-malignant cells and malignant cells lines were used to study the effect of the iron-chelators on mammalian cells. The exposure with the siderophores was done for 18–24 h because the average time of division for the mammalian cells in vitro is 18–28 h. Besides, siderophores are not respiratory inhibitors, and are expected to affect proliferation of cells. When some pathogenic bacteria were treated with these siderophores for 18–24 h, they were inhibited (manuscript under preparation). This was also taken into consideration whilst subjecting mammalian cells to siderophores for 18–24 h.

The experiments with mammalian cells showed that concentrations of Exo-MS and DFO-B that significantly inhibited the murine cancer cell line RAW 264.7 were not inhibitory to the normal cell line NIH/3 T3. It should be noted that both Exo-MS and DFO-B had no significant effect on the non-malignant human cell line HEK 293 up to 0.5 mg/mL and 1 mg/mL, respectively.

DFO-B also inhibited the proliferation of human breast cancer (MCF-7) and human leukemia (K 562) cell lines. However, it had no effect on human liver cancer cell line (HEPG2) even at 10 mg/mL. Exo-MS did not inhibit any of the human cancer cell lines tested. DFO-B has already been shown to have anti-proliferative effect on cancer cells [14], and hence was used here as a control.

When ferri-siderophores were evaluated for activity using the same MTT assay, no inhibitory effect on the cell proliferation was observed (data not shown). In this form, the siderophores were already saturated with iron, and therefore, could not deprive the cells of iron.

Earlier, some investigators have reported that a lipid-soluble siderophore from *Mycobacterium tuberculosis (Mtb)*, mislabelled by the authors as (desferri-)exochelin 772SM, induces death by apoptosis in human breast cancer cells without harming normal breast epithelial cells [15]. However, the correct term for the siderophore referred to by the authors should have been ‘Carboxymycobactin’, since water-soluble exochelin is not produced by *Mtb*.

Hoke et al. reported that DFO-B could be used as an adjunct to chemotherapy along with doxorubicin drug to inhibit breast tumor growth without any cardiotoxicity [16]. DFO-B was also found to be cytotoxic to malignant cells of neural origin [17, 18]. When DFO-B was given during the initial stages of tumor formation, it resulted in either regression or slower tumor growth due to decrease in intracellular Fe^3+^ concentration [19]. DFO-B was found to be inhibitory to leukemia cells in vivo as evident from the reduction in the cell numbers [20].

Since the water-soluble siderophores Exo-MS and DFO-B had no effect on HEPG2 even at high concentrations, effect of the lipid-soluble siderophore MBS was evaluated on HEPG2. MBS significantly decreased the percent survival of HEPG2 cells at very low concentrations (10–20 μg/mL). This concentration is almost 50–1000 times lower than Exo-MS and DFO-B concentrations that inhibited RAW 264.7 cell line. Due to its lipophilic nature, MBS may traverse eukaryotic cell membrane. This lipid-soluble characteristic makes MBS much more efficient at lower concentrations in trapping intracellular iron than the hydrophilic siderophores. Since very low concentrations of MBS showed anti-proliferative activity in vitro, it would be interesting to investigate its effects in vivo.

In the light of our results with the lipid-soluble MBS, conjugating water-soluble siderophores with lipid molecules may make them more effective against tumor cells. These siderophore conjugates could be evaluated as anticancer agents by themselves, or along with other therapies.

## Conclusions

These investigations provide a “proof of concept” that iron chelation therapy may be useful against malignant cells without any significant cytotoxicity on non-malignant cells. The anti-proliferative effect of siderophores on malignant cells needs further experimental and clinical studies to determine their role and utility in cancer treatment. The in-vivo toxicity of water-soluble siderophores may not be of concern, since DFO-B has already been approved by US-FDA for treating secondary iron-overload in patients suffering from thalassemia.

## Abbreviations

ACN: Acetonitrile
DFO-B: Deferoxamine-B
DMEM: Dulbecco’s Modified Eagle’s Medium
DPBS: Dulbecco’s Phosphate Buffered Saline
Exo-MS: Exochelin-MS
FBS: Fetal Calf Serum
HR-LC/MS: High-resolution liquid chromatography/Mass spectrometry
MBS: Mycobactin S
MTT: {3-(4, 5-dimethylthiazolyl-2)-2, 5-diphenyltetrazolium bromide}
NMR: Nuclear Magnetic Resonance
TFA: Trifluoroacetic acid
TOCSY: Total Correlation Spectroscopy

## References

[1] Hoffbrand AV, Ganeshaguru K, Hooton JW, Tattersall MH (1976). Effect of iron deficiency and desferrioxamine on DNA synthesis in human cells. Br J Haematol.

[2] Richardson DR (2002). Iron chelators as therapeutic agents for the treatment of cancer. Crit Rev Oncol Hematol.

[3] Cheng Z, Song Y, Xia J, Kang Y, Dai LL, Liu Y (2012). Effect of iron on the proliferation of lung adenocarcinoma cells in vitro. African J Pharm Pharmacol.

[4] Ratledge C, Hall MJ (1971). Influence of metal ions on the formation of mycobactin and salicylic acid in *Mycobacterium smegmatis* grown in static culture. J Bacteriol.

[5] Sharman GJ, Williams DH, Ewing DF, Ratledge C (1995). Isolation, purification and structure of exochelin MS, the extracellular siderophore from *Mycobacterium smegmatis*. Biochem J.

[6] Howard DH, Rafie R, Tiwari A, Faull KF (2000). Hydroxamate Siderophores of *Histoplasma capsulatum*. Infect Immun.

[7] Barclay R, Ratledge C (1983). Iron-binding compounds of *Mycobacterium avium, M. intracellulare, M. scrofulaceum*, and mycobactin-dependent *M. paratuberculosis* and *M. avium*. J Bacteriol.

[8] White AJ, Snow GA (1969). Isolation of mycobactins from various mycobacteria-The properties of mycobactins S and H. Biochem J.

[9] Ratledge C, Snow GA (1974). Isolation and Structure of Nocobactin NA, a Lipid-Soluble Iron-Binding Compound from *Nocardia asteroids*. Biochem J.

[10] MTT Cell Proliferation Assay – ATCC protocol, 2011. www.atcc.org/~/media/DA5285A1F52C414E864C966FD78C9A79.ashx.​

[11] Freshney RI (2000). Culture of Animal Cells Set. Med Clin (Barc).

[12] Ratledge C, Ewing M (1996). The occurrence of carboxymycobactin, the siderophore of pathogenic mycobacteria, as a second extracellular siderophore in *Mycobacterium smegmatis*. Microbiol.

[13] Ratledge C, Ewing DF (1978). The separation of the mycobactins from *Mycobacterium smegmatis* by using high-pressure liquid chromatography. Biochem J.

[14] Salis O, Bedir A, Kilinc V, Alacam H, Gulten S, Okuyucu A (2014). The anticancer effects of desferrioxamine on human breast adenocarcinoma and hepatocellular carcinoma cells. Cancer Biomark.

[15] Pahl PMB, Horwitz MA, Horwitz KB, Horwitz LD (2001). Desferri-exochelin induces death by apoptosis in human breast cancer cells but does not kill normal breast cells. Breast Cancer Res Treat.

[16] Hoke EM, Maylock CA, Shacter E (2005). Desferal inhibits breast tumor growth and does not interfere with the tumoricidal activity of doxorubicin. Free Radic Biol Med.

[17] Blatt J, Stitely S (1987). Antineuroblastoma Activity of Desferoxamine in Human Cell Lines. Cancer Res.

[18] Dayani PN, Bishop MC, Black K, Zeltzer PM (2004). Desferoxamine (DFO) – Mediated Iron Chelation: Rationale for a Novel Approach to Therapy for Brain Cancer. J Neuro-Oncol.

[19] Hann H-WL, Stahlhut MW, Rubin R, Maddrey WC (1992). Antitumor effect of deferoxamine on human hepatocellular carcinoma growing in athymic nude mice. Cancer.

[20] Estrov Z, Tawa A, Wang XH, Dube ID, Sulh H, Cohen A, Gelfand EW, Freedman H (1987). In vitro and in vivo effects of Deferoxamine in Neonatal Acute Leukemia. Blood.

